# The human gut virome: a multifaceted majority

**DOI:** 10.3389/fmicb.2015.00918

**Published:** 2015-09-11

**Authors:** Lesley A. Ogilvie, Brian V. Jones

**Affiliations:** ^1^School of Pharmacy and Biomolecular Sciences, University of BrightonBrighton, UK; ^2^Alacris Theranostics GmbHBerlin, Germany; ^3^Queen Victoria Hospital NHS Foundation TrustEast Grinstead, UK

**Keywords:** bacteriophage, human gut microbiome, metagenomics, virus-like particles, phage-encoded functions, dysbiosis

## Abstract

Here, we outline our current understanding of the human gut virome, in particular the phage component of this ecosystem, highlighting progress, and challenges in viral discovery in this arena. We reveal how developments in high-throughput sequencing technologies and associated data analysis methodologies are helping to illuminate this abundant ‘biological dark matter.’ Current evidence suggests that the human gut virome is a highly individual but temporally stable collective, dominated by phages exhibiting a temperate lifestyle. This viral community also appears to encode a surprisingly rich functional repertoire that confers a range of attributes to their bacterial hosts, ranging from bacterial virulence and pathogenesis to maintaining host–microbiome stability and community resilience. Despite the significant advances in our understanding of the gut virome in recent years, it is clear that we remain in a period of discovery and revelation, as new methods and technologies begin to provide deeper understanding of the inherent ecological characteristics of this viral ecosystem. As our understanding increases, the nature of the multi-partite interactions occurring between host and microbiome will become clearer, helping us to more rationally define the concepts and principles that will underpin approaches to using human gut virome components for medical or biotechnological applications.

## Introduction

In recent years it has become apparent that the microbes resident in and on us play a significant role in our health and well-being. These microbial residents have been shown to provide a range of essential functions, from helping us to digest food to conditioning our immune system, and providing protection from invading pathogens (Rakoff-Nahoum et al., 2004; Bäckhed et al., 2005; Ley et al., 2006; O’Hara and Shanahan, 2006; Zoetendal et al., 2006; Sokol et al., 2008). The advent of high-throughput sequencing technologies is enabling further insights into this complex human–microbe relationship, revealing significant associations between microbial ecosystem shifts and disease (termed dysbiosis), and bringing to light a diverse and abundant retinue of viruses intimately associated with the human gut microbiome. This human gut virome may be defined as the total population of viruses [or virus like particles (VLPs)] associated with the underlying gut microbial community. In keeping with the dominance of bacteria in the gut microbiome, the gut virome appears to be predominated by prokaryotic viruses (bacteriophage, or phage; Breitbart et al., 2003; Reyes et al., 2010; Minot et al., 2011). These encompass both DNA and RNA viruses that infect bacteria, but the latter are rare members of the phage family overall, and studies to date suggest these are accordingly a very minor component of the gut virome (Breitbart et al., 2003; Reyes et al., 2010; Minot et al., 2011).

Since the independent discovery of phages by Twort (1915) and d’Herelle (1917), the potential therapeutic and biotechnological application of these bacterial viruses has been a source of intense research interest. Phages are the most abundant infectious agents on the planet (>10^31^, extrapolated from Whitman et al., 1998), outnumbering their bacterial hosts, on average by 10:1 in most habitats studied (Wommack et al., 1999). Through their ability to lyse and kill host bacteria, phages play a crucial role in modulating bacterial community structure and function (Gorski et al., 2003; Brüssow et al., 2004; Suttle, 2005a,b, 2007; Rohwer and Thurber, 2009). This is perhaps most profoundly exemplified by the impact of phages on global biogeochemical cycles, and potentially the climate, exerted through effects on marine microbial ecology (Fuhrman, 1999; Breitbart, 2012; Brüssow, 2013).

Phages may also influence community function through the facilitation of gene transfer between strains and species (transduction), or themselves encode accessory functions of benefit to host species. Key functional attributes conferred from phages to their bacterial hosts include toxin synthesis, production of virulence factors, as well as genes that may provide metabolic flexibility (Fuhrman, 1999; Wommack and Colwell, 2000; Brüssow et al., 2004; Suttle, 2007). However, phage-host relationships are not static but dynamic co-evolutionary interactions, which play an important role in evolution of their bacterial hosts (Paterson et al., 2010). This repertoire of attributes and influences on bacterial hosts place phages as a potent force driving ecological functioning and evolutionary change in the prokaryotic world (Koskella and Brockhurst, 2014).

Phage (and virome) research is currently entering a renaissance, driven by an era of technological advancement that is seeing a shift from reductionist thinking. This advancement has made feasible investigation of complex microbial communities (such as the human gut microbiome) tractable on a systems level; a paradigm now increasingly extended to the viral components associated with such ecosystems (Reyes et al., 2012). Developments in high-throughput sequencing technologies and associated bioinformatics analyses are starting to provide crucial insights into the unique ecological characteristics of the human gut virome, shedding light on the structure and functioning of this viral collective. Recent work has revealed a surprisingly rich functional repertoire is encoded by the gut virome, with phages conferring a range of beneficial traits to their bacterial hosts that help maintain community stability, and afford resilience to invasion or disruption (Breitbart et al., 2003, 2008; Zhang et al., 2006; Reyes et al., 2010; Colomer-Lluch et al., 2011; Kim et al., 2011; Minot et al., 2011, 2012a,b; Modi et al., 2013; Ogilvie et al., 2013). Given this renewed interest and realization that human gut phages may play a significant role in shaping the development and functional outputs of host microbiomes, their potential for application in novel diagnostic, therapeutic, and biotechnological applications is now of considerable interest.

Here, we review recent insights into the human gut virome, focussing on the dominant phage component associated with this ecosystem, and examine currently available approaches to access, dissect, and elucidate the role of this viral collective. In doing so, we highlight the challenges to viral discovery and future routes of investigation.

## Life within the Human Gut

One of the most densely populated areas of the human body is the gastrointestinal tract (GIT), which provides a heterogeneous and expansive surface area (>200 m^2^) for microbial life (Hooper and Macpherson, 2010). The human gut is estimated to contain between 30 and 400 trillion micro-organisms (Suau et al., 1999), drawn from varied and abundant bacterial hosts that can support a correspondingly rich and varied phage population. Numbers of bacteria vary across the length of the GIT, with an enrichment of different bacterial types at different sites (Ahmed et al., 2007; Zhang et al., 2014). Corresponding studies of the variation in associated phage/virome populations have yet to be conducted in the human gut, although spatial analyses of the swine gut have revealed a differential distribution with increased abundance of phage encoded genes in the ileum, coinciding with reduced bacterial numbers (Looft et al., 2014). These observations have been linked to gut physiology. Due to the cycle of feast and famine likely occurring in the ileum (as food is ingested and processed), and the role of phages in nutrient release through lysis of host bacteria, there could be fitness costs associated with phage resistance in ileal bacteria (Looft et al., 2014). The physical and physiochemical heterogeneity is further enhanced by a thick mucosal layer that separates the intestinal epithelium from the lumen. This highly dynamic mucosal layer is in constant flux, acting both as a protective barrier to invading pathogens and residence for commensal bacteria and their attendant viral collective (Barr et al., 2013a,b). The physical conditions likely created by this mucous layer will undoubtedly influence the nature of the interactions which occur between the human host, bacteria, and phage. Indeed, enrichment of phage numbers within mucosal environments as well as elevated ratios of phages to bacteria have been reported (Barr et al., 2013a), as well as the potential role of these phage and the mucosal environment in the establishment of a non-host derived phage based innate immunity (Barr et al., 2013a).

## Human Gut Virome Characteristics

In many environments phages have been found to outnumber their bacterial hosts by an order of magnitude (Brüssow and Hendrix, 2002; Suttle, 2005a). In the human gut, however, phages are believed to exist at levels comparable to their bacterial hosts. Based on microscopy counts, bacteria appear typically at ∼10^9^/g feces and VLPs ∼10^8^–10^9^/g (Kim et al., 2011), with most VLPs originating from phages (Reyes et al., 2010; Minot et al., 2011). VLP-derived estimates of phage diversity, in terms of phylotypes, also reflect that of bacterial hosts, with estimated ratios of 1:1 (phage, bacteria; Reyes et al., 2010; Minot et al., 2011). Similar to their bacterial hosts, phages have been found to accumulate on gut mucosal surfaces and within mucous, but here are found at much higher levels with phage, bacteria ratios of approximately 20:1 within the mouse intestinal mucosa (Barr et al., 2013a).

In contrast to non-host associated ecosystems such as aquatic environments, predator-prey interactions or ‘kill-the-winner’ phage-host dynamics (Brüssow and Hendrix, 2002; Thingstad et al., 2008), are seemingly lacking in the human gut microbiome (Reyes et al., 2010). Instead, most of the dominant virotypes detected in the gut ecosystem show evidence for a temperate lifestyle (in which phages integrate into host chromosomes or exist as quiescent episomal elements at the expense of lytic replication), as indicated by the frequency of integrase genes and other genetic features (Breitbart et al., 2003; Reyes et al., 2010; Minot et al., 2011). The genetic symbiosis established by temperate phage, persisting as prophage within host cells, is important for genetic exchange between bacterial hosts, alteration of host phenotypes *via* lysogenic conversion (Brüssow et al., 2004; Reyes et al., 2010), which in turn impacts on bacterial host fitness as well as human gut microbial dynamics (Thingstad et al., 2008; Duerkop et al., 2012). In this respect, the carriage of phages as dormant prophage may still have profound implications for the competitive abilities of bacterial hosts, potentially ensuring dominance of their intestinal niche in the presence of closely related competing strains (Thingstad et al., 2008; Duerkop et al., 2012).

These observations do not exclude the possibility that other phage life cycles and strategies exist. Intriguing observations within the oral virome (Abeles et al., 2014) have suggested that phage could also be lysing hosts at a constitutive low level, with longitudinal analyses of the oral viromes of eight human subjects consistently identifying specific phages at all time points over a 60-days period and in all subjects sampled (the specific phage detected varying between individuals); while observations of phages accumulating within mucosal environments (Barr et al., 2013a) open up the possibility of a phage survival strategy, with the mucosa facilitating phage persistence until a suitable host is encountered (Edlund et al., 2015). Whether the same holds true for the human gut is a question still to be answered.

Although human gut phages are often found to have high specificity to host bacterial species and even strains *in vitro* (e.g., Weinbauer, 2004; Ogilvie et al., 2012b), this phenomena may only be an artifact of the laboratory setting (Chibani-Chennoufi et al., 2004). Observations of phage host range expansion following antibiotic exposure within the mouse gut (Modi et al., 2013) highlight the adaptive capacity and potential genetic plasticity of phages (Chibani-Chennoufi et al., 2004), but nevertheless *in vivo* confirmation of phage host range and specificity within the human gut is severely lacking.

The human gut virome is also seemingly characterized by a high degree of inter-personal variation (much higher than the associated gut microbiome) and remarkable temporal stability in structure (Reyes et al., 2010; Minot et al., 2011, 2013). Reyes et al. (2010) report retention of >95% of viral genotypes within one individual, with minimal changes in relative abundances, over a 1 year period. Similarly, Minot et al. (2011) report persistence of 80% of virotypes within an individual over a 2.5 years period. The observed high level of inter-individual variation has been linked to variation in the bacterial component of the human gut microbiome carried by individuals, which has been found to vary at the level of species and strain (Huttenhower et al., 2012), persistence of a small portion of the global virome within each individual, and also the ability of viral populations (in particular lytic phage) to undergo rapid evolution to form new “species” of phages (Minot et al., 2013). Greater similarity between viromes is found, however, when individuals eat the same diet (Minot et al., 2011), likely reflecting dietary induced convergence also found in their bacterial hosts (Wu et al., 2011). Moreover, recent studies, mainly based on bioinformatics-based interrogation of whole community metagenomes, are demonstrating that there may be a higher level of virotype conservation between individuals than previously thought (Stern et al., 2012; Ogilvie et al., 2013; Dutilh et al., 2014).

Many of these assumptions have, however, been made based on the analysis of a relatively limited number of individuals. In comparison, many of the studies investigating the diversity of the human gut bacterial component of the gut ecosystem, i.e., based on the analysis of 16S rRNA sequences, involve study numbers of generally an order of magnitude or more. In the future, a more detailed interrogation of the human gut virome over a wider range of temporal and spatial scales and comprising larger cohorts is required to fully delineate the characteristics of this viral ecosystem.

## Accessing and Analyzing the Human Gut Virome – Challenges and Solutions

Our current state of knowledge of the human gut virome has been informed by a combination of traditional culture-based techniques, high-resolution microscopy, and metagenomic analyses. Taking an analogous trajectory to that of characterizing the bacterial component of human gut microbiome, approaches to sampling the associated complex viral communities populating this ecosystem have moved from reliance on more traditional culture and microscopy-based methodologies, toward metagenomic analysis of nucleic acids by next generation sequencing technologies. **Table 1** provides an overview of current approaches commonly used for accessing and understanding the human gut virome.

**Table 1:** Benefits and limitations of commonly used methods for accessing the human gut virome.

Method	Description	Benefits	Limitations
**Culture-based**
– Plaque-based assays	Isolation of phages from a biological sample using a cultured bacterial host	• Essential tool that has underpinned our understanding of phage-host interactions	• Culture dependent
		• Bacterial host is known	• Limited range of bacterial hosts, due to inability to culture >99% of bacteria
		• Host–phage dynamics can be investigated in real time	• Recapitulation of *in vivo* observations *in vitro* is questionable
		• Inexpensive
		• Accessible for most laboratories
		• Amenable to testing hypotheses
**Microscopy**
– Transmission electron microscopy (TEM)	Visualization of viruses following negative staining	• Culture independent	• No functional information • Little or no community level insight
		• Provides *in situ* high resolution structural visualization (TEM) of viruses	• No host range information unless used to view purified phages propagated on a culturable bacterial host
		• Provides information of morphological diversity of viral/phage types	• Requires high virus like particle/phage titres for visualization
		• Useful as supplement to other approaches	• Phages with unusual capsid structures, e.g., Inoviridiae, may be difficult to identify
– Fluorescence	Visualization of viruses following staining by DNA fluorochromes	• Culture independent	• As for TEM, but additionally, information on viral structure and morphology is not provided, with information largely restricted to viral numbers
		• Quantitative
		• Rapid
		• Cost effective
		• Broad overview of viral numbers
**PCR-based**
– PCR/quantitative PCR	• Amplification of DNA segments using primers targeting a specific genomic section	• Culture independent • Quantitative	• Lack of conserved genes within all phages akin to the 16S rRNA gene in bacteria
			• View of viral/phage abundance and diversity limited to available sequence data from which primers are designed
**Metagenomics**
– Large/small insert metagenomic libraries	• Isolation of nucleic acids from virus like particles followed by direct cloning in surrogate host species	• Culture independent	• Potential bias in inventory gained as focuses on free viral particles (i.e., actively replicating phage)
		• Can provide access to accessory genes and potential assessment of function/impact on host bacteria	• Laborious and expensive, with insights limited by scale of libraries that can be generated and screened
		• Can provide community-level insights	• Biases due to difficulties in cloning phage DNA and maintaining in surrogate host may be apparent
			• Use of single surrogate host may limit heterologous gene expression and therefore utility in functional evaluation of phage genes
			• Loss of host range information
– Virus-like particle libraries	• Isolation of virus like particles from biological samples using centrifugation and/or filtration methods, extraction, and amplification of encapsulated DNA followed by high-throughput sequencing	• Culture independent	• Potential bias in inventory gained as focuses on free viral particles (i.e., actively replicating phage)
		• In-depth inventory of composition and functional repertoire of viruses within biological samples	• Viral DNA recovered can be very low therefore amplification techniques used that may bias community snapshot gained
		• Assessment of viral diversity and lifestyles within the human gut	• No host information
		• Community-level assessment	• Large fraction of recovered sequences are ‘unknown’
			• Potential contamination of VLP libraries with bacterial DNA
			• Relatively expensive for routine/repeated analyses
			• Difficult to test hypotheses generated
			• Loss of host range information
– Whole community shotgun sequencing	• Extraction of total DNA from a biological sample, followed by library preparation and high-throughput sequencing	• Viral DNA is significant fraction of extracted DNA	• Difficult to distinguish between bacterial and viral sequences
		• Full microbial community snapshot gained	• Identification of host species remains challenging
		• Community level inventory	• Potential bias in inventory gained as focuses on ‘quiescent’ fraction of virome
		• Structural and functional information provided	• Relatively expensive for routine/repeated analyses
			• Difficult to test hypotheses generated

Culture-based techniques involving the isolation of phages from the environment using a specific bacterial host have informed our basic understanding of phages for decades and will continue to be an essential tool for human gut viral discovery. Indeed, our present knowledge of phage-host interactions, functional repertoire, and phage ecology is largely underpinned by numerous detailed *in vitro* analyses based on conventional plaque assays used to isolate phages and associated virology. Such techniques are inherently limited by the inability to culture >99% of bacterial species found in many environments (Amann et al., 1995; Rappé and Giovannoni, 2003), however, ongoing efforts are substantially increasing representation of culturable gut inhabitants (Rajilić-Stojanović and de Vos, 2014). There is also uncertainty regarding the degree to which observations generated under highly controlled, standardized, and essentially artificial laboratory conditions reflect the situation *in vivo.* This is particularly true for understanding the functioning of complex communities such as the gut microbiome where, although there are some good systems available that replicate some of the features of this system, it is still difficult to develop realistic and representative *in vitro* models.

High resolution microscopy analysis techniques such as transmission electron microscopy (TEM) have also proved essential for the study of phage structure and identification of phage types with distinct morphologies. Prior to electron microscopy the structure and basic nature of viruses in general was a complete unknown, with their existence known only through observations that for some diseases, cell free tissue extracts remained capable of causing the original disease. This provided the origin for the term virus, which was derived from the latin word describing a poisonous or venomous substance. Electron microscopy has also provided early insight into the composition of the human gut viral community based on phage structure and morphology, which continue to be important parameters in viral classification systems. Such studies revealed tailed phages (Order *Caudovirales*) to be prevalent (Flewett et al., 1974; Letarov and Kulikov, 2009); observations corroborated and expanded by more recent metagenomic-based catalogs, showing a dominance of tailed, doubled stranded DNA viruses of the order *Caudovirales* (*Siphoviridae, Myoviridae, Podoviridae*) alongside the tail-less single stranded DNA viruses (*Microviridae*; Reyes et al., 2010; Kim et al., 2011).

Recent rapid progress in sequencing technologies and associated bioinformatics methodologies has enabled a more in-depth view of the structure and functioning of these viral communities. Development of methods to isolate VLPs from fecal material using a combination of filtration and density gradient ultra-centrifugation techniques, followed by extraction and amplification of viral nucleic acids and subsequent sequencing (Thurber et al., 2009; Reyes et al., 2012), have provided unprecedented glimpses into the viral fraction of the human gut ecosystem, providing the first in-depth inventory of the composition and functional repertoire of this collective (Breitbart et al., 2003; Reyes et al., 2010; Kim et al., 2011; Minot et al., 2011).

In keeping with studies of the bacterial fraction, such metagenomic approaches have now become the mainstay of viral ecology and are set to usher in a new era of viral ecogenomics akin the renaissance recently enjoyed by microbial ecology. There remain, however, a number of challenges associated with the use and interpretation of results generated using metagenomic techniques. Inherent in the extraction of VLPs is a focus on the analysis of free and therefore actively replicating phage particles present at the time of sampling, restricting access to the quiescent fraction. In addition, amplification procedures implemented due to low levels of extracted DNA, potentially exclude the analysis of certain phage types (Angly et al., 2006), biasing the snapshot of the virome gained from these methods. However, perhaps most important for community level ecogenomic investigations is the lack of host range information afforded by metagenomic approaches. Although the culture-independent nature of these methods is a key advantage, this effectively divorces the resulting sequence data from bacterial host-range information, necessitating the use of additional bioinformatic approaches to infer this indirectly.

**FIGURE 1:**
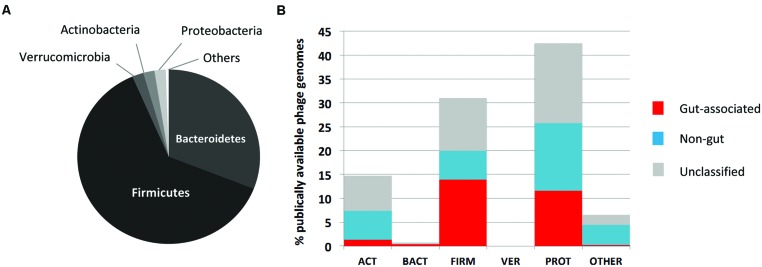
**Overview of available human gut phage genomic sequences. (A)** Major phylogenetic divisions of bacteria within the human gut microbiome. This community is dominated by members of the Bacteroidetes and Firmicutes. **(B)** Distribution of 611 phage genomes available on NCBI by phylogenetic division of host bacteria, and association with the human gut. Phages have been classified as gut associated (red bars), non-gut (blue bars) or remain unclassified (gray bars); based on Blastn searches of 168 metagenomic data sets of human or environmental origin. Adapted from Ogilvie and Hirsch (2012).

Our understanding of the functional and phylogenetic make-up of the human gut phage/viral gene space has largely been based on alignment-based techniques such as the Blast suite of algorithms (Altschul et al., 1990), exploiting homologies between sequences at the nucleotide and amino acid levels. These methods have also highlighted the vast level of novel gene content encoded by phage genomes, evident in the lack of homology of many phage genes to existing database entries (Breitbart et al., 2003; Reyes et al., 2010; Minot et al., 2011). Such novelty poses a significant challenge to efforts seeking to understand the basic structure and function of viral communities, and is compounded further by the dearth of well-characterized phage reference genome sequences derived from those viruses established to infect key members of the gut microbiome (**Figure 1**). This is exemplified by the lack of genomic data on phages infecting *Bacteroides* spp., prominent human gut members and implicated in both the onset of and protection against inflammatory-mediated disease (Wexler, 2007; Mazmanian et al., 2008), with only two representative genomes (e.g., Hawkins et al., 2008; Ogilvie et al., 2012b) currently (March 2015) deposited in global sequence databanks. On a more general level, estimates suggest less than 0.001% of the predicted phage diversity is represented in global sequence databanks (Bibby, 2014). In contrast, the interpretation of metagenomic data derived from the bacterial components of the gut ecosystem has benefitted greatly from access to an ever expanding catalog of complete genome sequence data from diverse organisms and habitats.

Evolutionary and ecological insight into the gut virome has also been hampered by the lack of a common phylogenetic anchor in phages (akin to the 16S rRNA gene in bacteria), enabling detailed studies of the phylogenetic relationships existing between ecosystem members. Although conserved phage genes do exist, such as the terminase gene (Casjens, 2003), the significant mosaicism exhibited by phage genomes obscure any robust phylogenetic signal.

Whole-community metagenomes, generated from DNA directly extracted from fecal material and containing significant fractions of viral DNA (4–17%, Qin et al., 2010; Minot et al., 2011), are now being considered as a valuable resource for the analysis of phage communities that when placed in tandem with VLP-derived datasets have the potential to provide a more cohesive overview of the phage component of ecosystems, including the human gut. Despite these potential benefits, both VLP-derived and whole community metagenomic datasets present a number of challenges for analysis of the data from a viral perspective. In particular, distinguishing between viral and bacterial sequence reads, allocating reads to distinct genomes, and assigning host species to identified phage. As noted above, these challenges are further compounded not just by the diversity of the virosphere, but also the lack of complete and well-characterized phage reference genomes with established host range and functionality.

To overcome some of these hurdles a range of inventive and insightful viral-orientated bioinformatics tools, pipelines, and approaches have emerged in recent years, which are aimed at standardizing and improvement of assembly, annotation, and comparative analysis of viral metagenomes. These include VIROME (Viral Informatics Resource for Metagenome Exploration; Wommack et al., 2012), MetaVir (Roux et al., 2014), iVireons (Seguritan et al., 2012), and PHACCS (PHAge Communities from Contig Spectrum; Angly et al., 2005). Application of these viral/phage-orientated analysis methodologies are starting to heighten our understanding of this novel gene space.

Stern et al. (2012) exploited the anti-phage immune system of bacteria, the CRISPR (clustered regularly interspaced short palindromic repeats)/Cas (CRISPR-associated) system, to identify phage sequences within human gut microbial metagenomic datasets. The CRISPR/Cas system represents a mechanism of bacterial acquired adaptive immunity against phage, based on a genome-based catalog of previous infections. The CRISPR system comprises short repeated sequences that are separated by hypervariable CRISPR spacers of between 24 and 50 bp. Bacteria incorporate DNA of the infecting phage into these spacers, effectively establishing a genome-based phage resistance catalog, which when transcribed into small RNA and in tandem with formation of a Cas protein complex, block the replication of similar phages (Sorek et al., 2008). The CRISPR spacers identified by Stern et al. (2012) within raw sequencing reads of 124 whole community metagenomes (Qin et al., 2010), were used to probe assembled sequence contigs; this resulted in detection of approximately 1000 partial and complete phage genomes, which appeared in multiple individuals of diverse geographic origin (Stern et al., 2012). Bacterial hosts for some of these phage were identified and insight into patterns of phage-bacteria co-existence was gained. Results which pointed to the existence of a reservoir of phage frequently associated with the gut microbiome, but no obvious evidence for the existence of similarities in gut viromes, or enterotypes, as posited for the microbial component (Arumugam et al., 2011).

Taking advantage of similarities in global nucleotide usage patterns, or the genome signature, arising between phages infecting the same or related host bacterial species (Pride et al., 2003, 2012), a genome-signature based approach has also been developed for the phage-orientated dissection of conventional whole-community metagenomes (Ogilvie et al., 2013). Comparison of tetranucleotide usage profiles (patterns of four nucleotides) exhibited by a well-characterized set of phage genomes with known host-range, were compared to 139 human gut microbial metagenomic datasets using TETRA (Teeling et al., 2004). Sequences exhibiting similar tetranucleotide frequency patterns were identified, retrieved and functionally profiled to identify sequences of phage origin, infer host range and provide insights into phage lifestyles within the human gut. Using this approach a portion of the human gut virome poorly represented in VLP-derived metagenomes was revealed, illuminating phylogenetically distinct, predominantly temperate, phage genomes encoding a range of functions relevant to human health (Ogilvie et al., 2013). Furthermore, shared patterns of distribution in multiple individuals were identified indicating the existence of putative viral enterotypes (Ogilvie et al., 2013), and providing continuity with the ever expanding knowledge base on bacterial constituents of the human gut microbiome (Arumugam et al., 2011).

Most recently, application of a *cross-assembly* method (Dutilh et al., 2012, 2014) – a bioinformatics approach that assumes sequences repeatedly found in different metagenomes are most likely to be part of the same genome – for the reanalysis of 12 existing human gut virome datasets (Reyes et al., 2010), revealed the existence of a highly abundant 92 kb phage in multiple individuals (Dutilh et al., 2014). Interestingly, these most recent publications demonstrating alternative approaches to the re-analysis of human gut microbiomes and viromes (Stern et al., 2012; Ogilvie et al., 2013; Dutilh et al., 2014), challenge the notion that our gut viromes are highly individualized due to the perceived fast-paced evolutionary race with their bacterial hosts, and are perhaps surprising in light of the vast genetic diversity reported in many ecosystems.

While these collective observations are seemingly contradictory to studies focusing on the generation and analysis of VLP based metagenomes, it should be noted that they are based on using datasets generated using distinct approaches and therefore may afford distinct insights into the gut virome not possible with VLP based datasets. Unlike the bacteria-centric metagenomes used in the studies noted above (Ogilvie et al., 2012a; Stern et al., 2012; Dutilh et al., 2014), VLP-based libraries are focused instead on actively replicating “free” phage at the time of sampling. In contrast analysis of whole community metagenomes provide access to the quiescent fraction, i.e., phages that may be inactive or dormant due to lysogenisation and poorly represented or absent from VLP-derived datasets, as noted in genome signature based dissection of gut metagenomes (Ogilvie et al., 2013). Such phages may nevertheless make important functional contributions to the community in terms of providing bacterial hosts with accessory functions, and potentially also resistance to infection by related phage types. Such observations highlight the need for complementary approaches to accessing the human gut virome, if the entirety of this viral ecosystem is to be cataloged and understood.

## The Human Gut Phage Functional Landscape

Within marine environments phage-encoded genes are known to make a significant impact on bacterial-mediated ecosystem functioning (Fuhrman, 1999; Suttle, 2005a,b), but the contribution of phages to the human gut microbial ecosystem is less well-established. The functional landscape of the human gut virome is, however, beginning to be mapped, revealing a surprisingly rich functional repertoire. Recent metagenomic surveys have revealed that gut associated phages encode genes that are generally beneficial for intestinal bacteria, including functions that help host bacteria adapt to their environment, relate to bacterial virulence, and functions that help maintain host microbiome stability and community resilience (Breitbart et al., 2003, 2008; Zhang et al., 2006; Reyes et al., 2010, 2012; Colomer-Lluch et al., 2011; Kim et al., 2011; Minot et al., 2011; Duerkop et al., 2012; Modi et al., 2013; Ogilvie et al., 2013).

Analysis of VLP libraries has revealed an abundance of genes involved in energy harvest, e.g., carbohydrate and amino acid metabolism (Reyes et al., 2010; Minot et al., 2011), including the BACON domain (Reyes et al., 2013; Dutilh et al., 2014), with potential roles in targeting glycoproteins and possibly host mucin (Mello et al., 2010; Reyes et al., 2013). These findings suggest that phages could be playing key roles in human host metabolism through indirect effects on their bacterial hosts. Whereas cryptic prophages (i.e., devoid of genes required for lytic development) have been shown to be essential to their bacterial hosts (*Escherichia coli*), enabling them to withstand adverse conditions, such as antibiotic, osmotic, oxidative and acid stresses, increasing growth and influencing biofilm formation (Wang et al., 2010; Reyes et al., 2013).

Phages may also constitute a repository of ‘beneficial’ genes in the gut microbiome, safeguarding important activities and genetic information during adverse advents that lead to the disruption of the community, and its subsequent re-establishment. Global analysis of the gut virome in mice revealed that following exposure to antibiotics, gut phage populations were enriched for genes associated with antibiotic resistance, as well as those related to gut colonization, and growth and adaptation of their microbial hosts in this environment (Modi et al., 2013). In this scenario, the virome was theorized to provide a functional buffer of genes necessary for community recovery following exposure to agents such as antibiotics.

The phage-encoded antibiotic resistance gene pool within the human gut is significant and diverse (Reyes et al., 2010; Minot et al., 2011) and has been shown to be viable, mobile, and widely distributed within multiple individuals of diverse geographical origin (Modi et al., 2013; Ogilvie et al., 2013). These observations reinforce the notion that phages may play a more significant role in the emergence and spread of antibiotic resistance genes than previously thought (Ogilvie et al., 2013). Given that phages have been shown to move between biomes (Breitbart et al., 2004; Sano et al., 2004; Breitbart and Rohwer, 2005), and the recognition that phages are a reservoir of antibiotic resistance determinants, both within and out-with the human gut (Colomer-Lluch et al., 2011; Modi et al., 2013; Marti et al., 2014; Muniesa and Jofre, 2014), understanding of the factors that result in mobilization of phage-encoded functions may be crucial to develop strategies that limit the spread of, e.g., antibiotic resistance and the emergence of pathogenic bacterial strains.

Overlaying these more direct functional attributes or contributions of gut-associated phage, is their involvement in wider mechanisms of genetic transfer. An extensive network of gene exchange exists within the human microbiome as a whole; with the human gut estimated to exhibit one of the highest rates of transfer of all body sites analyzed (Smillie et al., 2011). The observed or estimated rate of gene exchange is undoubtedly influenced by the environmental and physiological parameters within the human gut milieu (Licht and Wilcks, 2005), but as part of the mobile metagenome (Jones and Marchesi, 2007; Jones, 2010; Ogilvie and Jones, 2013), phages likely play a key role in this network through the process of transduction (generalized or specialized); a process in which DNA is transferred from one bacterium to another. Given the abundance of phages in the gut microbiota, and that this community is essentially comprised of high numbers of closely related strains and species, it is conceivable such a mechanism could be active within the human gut, exemplified by the phage-mediated transduction of plasmids between *Lactococcus lactis* and *Streptococcus thermophilus* (Licht et al., 1999). Overall, the human gut phage functional repertoire, similar to other mobile genetic elements such as plasmids, aligns with challenges and activities important to life within the human gut (Jones and Marchesi, 2007; Jones, 2010; Ogilvie et al., 2012b; Ogilvie and Jones, 2013). Moreover, as the full extent of the role of phages as agents of horizontal transfer with the human gut is revealed, their impact on host functional outputs will become clearer, with potential implications for human health and well-being.

## An Emerging Role for Gut Phages in Health and Disease

In recent years there has been intense interest in characterizing the bacterial dysbiosis signature of disease, with alterations in the structure and functioning of the human gut microbiome being associated with many conditions from inflammatory bowel disease (IBD) and colorectal cancer, to diseases of the central nervous system (e.g., Sobhani et al., 2011; Clemente et al., 2012; Cryan and Dinan, 2012; Wang et al., 2012; Ohman et al., 2015). Nascent observations are now starting to link the associated retinue of phages with the pathogenesis of disorders associated with perturbation of the gut ecosystem (Furuse et al., 1983; Lepage et al., 2008; Pérez-Brocal et al., 2013; Wagner et al., 2013; Dinakaran et al., 2014; Norman et al., 2015).

Early culture-based studies revealed quantitative and qualitative differences in phages isolated from fecal samples derived from healthy individuals and those with leukemic and internal disorders, with generally higher levels of coliphages in patient samples as compared to healthy subjects (Furuse et al., 1983). Lepage et al. (2008) used epifluorescence microscopy to detail the abundance of VLPs within gut mucosal samples taken from healthy and diseased individuals, revealing an increased number of VLPs in individuals suffering from Crohn’s disease (CD), observations supported more recently by other metagenomics based studies revealing an altered representation of phages in individuals with CD compared to healthy controls (Wagner et al., 2013) and increased enteric virome richness and decreased bacterial diversity in individuals suffering from CD and ulcerative colitis (Pérez-Brocal et al., 2013).

Deep sequencing of blood samples (plasma DNA) has also provided some evidence of altered phage taxonomic and abundance profiles associated with host bacterial species, within patients suffering cardiovascular disease (Dinakaran et al., 2014). In the latter study, altered phage composition is hypothesized to result from microbial translocation across the intestinal barrier, which becomes more permeable following cardiac surgery due to gut ischemia (narrowing or blockage of blood vessels). Such observations, although preliminary, lead to the question of what role human gut phages are playing in specific diseases, and in particular the microbial dysbiosis often associated with a range of diseases.

Barr et al. (2013a) recently posited a key role for phages in human health, suggesting that phages residing in metazoan mucosal surfaces provide a non-host derived anti-microbial defense. The model suggests that metazoan mucosal surfaces and phages coevolve to maintain phage adherence. This benefits the metazoan host by limiting mucosal bacteria, and benefits the phage through more frequent interactions with bacterial hosts. The study revealed that in divergent animal mucosa, phages were found to accumulate at significantly higher densities in comparison to non-mucosal surfaces. The observation that phage abundance is high in mucosal surfaces is of course not ground-breaking in itself and could simply be explained through increased replication of phages due to the higher densities of bacterial hosts they encounter in these mucosal environments. Barr et al. (2013a), however, showed that even in the absence of host bacteria, phage abundance was greater on cells producing mucus in comparison to non-mucus producing cells. The authors also ruled out the likelihood that the accumulation of phage was simply the result of the laws of mass-action, i.e., the gel-like properties of the mucus itself slowing the diffusion of phage. Finally, the authors showed that enrichment of phage on mucosal surfaces was occurring via interactions between host mucin glycoproteins and phage immunoglobulin-like protein domains exposed on phage capsids. These observations suggest a symbiotic relationship between phages and metazoan hosts that provides a previously unrecognized antimicrobial defense that actively protects mucosal surfaces (Barr et al., 2013a). It must be noted, however, that the study by Barr et al. (2013a) did not involve human gut samples. Whether or not such a symbiotic relationship exists within the human gut has still to be shown. Moreover, other potential roles of phage within mucosal layers cannot be excluded, such as a survival strategy allowing phage to persist for long periods (in the absence of producing virions), until a suitable host is found (Edlund et al., 2015).

**FIGURE 2:**
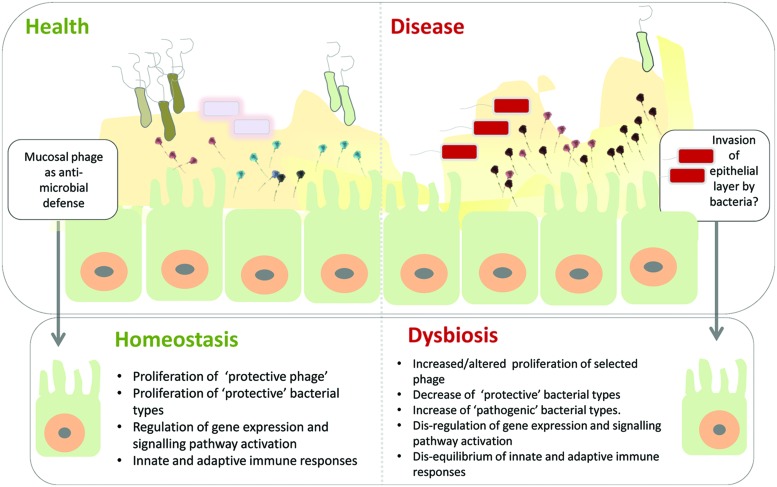
**Model of phage mediated-dysbiosis.** Genetic and environmental factors combine to present a gastrointestinal mucosal environment which modifies the adherent phage community. The anti-microbial properties of these mucosal phages – bacteriophage adherence to mucus (BAM) model proposed by Barr et al. (2013a) – are altered compared to healthy individuals leading to microbial dysbiosis, e.g., via proliferation of pro-inflammatory bacterial types and/or depletion of selected protective bacterial types. Invasion of the epithelial cell layer by bacteria may create a cellular environment in which the complex microbial-epithelial interactions are disrupted resulting in, e.g., alterations in gene expression and signaling pathway activation as well as disequilibrium of innate and adaptive immune responses. Top panel of figure inspired by Barr et al. (2013a).

With these caveats in mind it is still tempting to speculate and hypothesize that disruption of this symbiosis may be a significant component of the microbial dysbiosis signature associated with a range of human diseases. In this scenario the human host genetic background and/or lifestyle factors may lead to an altered representation of anti-microbial mucosal phage, e.g., increased abundance of phages leading to selected reduction in bacterial species, potentially propelling the microbial community into dysbiosis, leading to deregulation of protective microbial-epithelial cell interactions (Hooper and Gordon, 2001; Kostic et al., 2013). Such deregulation of the complex and finely tuned interactions associated with intestinal immune responses could potentially allow invasion of ‘dysbiotic’ bacterial intruders through the epithelial cell layer (Hooper and Gordon, 2001; Kostic et al., 2013; see **Figure 2**). Realization that phages may contribute to a non-host derived immunity, provides a range of possibilities to modulate this mucosal phage complement through targeted interventions to prevent and or treat intestinal diseases (see also Barr et al., 2013b).

## Future Outlook

With advances in high-throughput sequencing technologies and associated methods for sequence analysis and assignment, we are now starting to gain some crucial insight into the unique ecological characteristics of the human gut virome, shedding light on its structure and functioning. Although not without limitations, the application of these novel methodologies is providing the potential to gain unprecedented access to this human gut collective, allowing us to further explore the complex nature of the phage-host relationship. Concepts and principles that will aid our understanding of this environment are in the process of being formed, shaped by incremental increases in knowledge that place gut phages on a continuum from simple gene transfer agents to community level functional buffers, and potential co-regulators of human immune defense systems and drivers of ecosystem diversity.

It is evident that viral metagenomics faces many major challenges, not least the issues of sequence analysis and classification, but current solutions and approaches to analyzing these datasets are widening our perspectives on human gut ecology at a rapid pace. We must, however, proceed with caution, and heed the lessons learned from microbial ecology over the last decade. Wholescale investment in metagenomic approaches, powerful as they are, at the expense of the established more traditional-culture based methods, is unlikely to deliver the full story and we must not progress in one methodology at the expense of skills and capacity in underpinning basic virology. A combination of approaches, including emerging approaches for studying phage-host interactions (Dang and Sullivan, 2014), is necessary for a complete understanding and, perhaps most importantly, for application of knowledge gained.

With new insights will also come more questions. The suggestion that gut mucosal phages could enter into a mutualistic relationship with their human host directly (without working through the indirect manipulation of community structure and output), essentially suggests the existence of a hitherto uncharacterized branch of the human immune system (Barr et al., 2013a). Further proof of such functionality may actually force us to further refine our understanding of the inherent ecological characteristics of the gut ecosystem (as well as our assessment of components comprising the human holobiont), and opens for manipulation an additional range of complex interactions between human host, microbiome, and virome that may be used to enhance human health and well-being. Without doubt, further understanding of the role of human gut-specific phages within healthy and diseased individuals is crucial to the successful harnessing of the full diagnostic and/or therapeutic potential of the human gut virome.

## Conflict of Interest Statement

The authors declare that the research was conducted in the absence of any commercial or financial relationships that could be construed as a potential conflict of interest.
